# Effects of flavophospholipol on conjugation and plasmid curing of multidrug-resistant *Salmonella* Enteritidis in broiler chickens

**DOI:** 10.1093/jacamr/dlab022

**Published:** 2021-03-11

**Authors:** Kelvin Lim, Michael Pennell, Stephanie Lewis, Mohamed El-Gazzar, Wondwossen A Gebreyes

**Affiliations:** Veterinary Health Management Branch, National Parks Board, 6 Perahu Road, Singapore 718827; Division of Biostatistics, College of Public Health, The Ohio State University, 1841 Neil Ave, 246 Cunz Hall, Columbus, OH 43210, USA; Animal Resource Center, University of Texas Southwestern Medical Center, 5323 Harry Hines Blvd., Dallas, TX 75390, USA; Department of Veterinary Diagnostic and Production Animal Medicine, Iowa State University, 2203 Lloyd Veterinary Medical Centre, Ames, IA 50011, USA; Department of Veterinary Preventive Medicine, College of Veterinary Medicine, The Ohio State University, A100R Sisson Hall, 1920 Coffey Rd, Columbus, OH 43210, USA

## Abstract

**Background:**

Early *in vitro* studies suggested that flavophospholipol has plasmid-curing effects and could inhibit conjugation by disrupting pilus formation between bacteria.

**Objectives:**

This 36-day controlled-challenge study aimed to evaluate the anti-conjugative and plasmid-curing effect of flavophospholipol *in vivo* on plasmid-mediated antimicrobial resistance (AMR) in MDR transconjugant *Salmonella* Enteritidis in chickens.

**Methods:**

A total of 270-day-old chicks were randomly assigned to four control and four treatment groups with two doses of in-feed flavophospholipol (10 ppm and 64 ppm) and in the presence and absence of ampicillin in drinking water. Chicks were orally challenged with *Salmonella* Enteritidis with known plasmid-encoded AMR factors. Cloacal swabs were collected on Day 7, 14 and 23. On Day 35, all chickens were euthanized, and caecal tissue and content were collected. Antimicrobial susceptibility testing was done with a panel of 12 antimicrobials and interpreted according to CLSI breakpoints.

**Results:**

Flavophospholipol given in-feed at 64 ppm had an anti-conjugative effect. There was a significant reduction of acquisition of resistance to ampicillin, streptomycin and tetracycline by the recipient strains of *Salmonella* Enteritidis in treatment groups given flavophospholipol in-feed at 64 ppm (*P < *0.05). This was not seen with flavophospholipol given in-feed at 10 ppm.

**Conclusions:**

The results demonstrate that flavophospholipol given in-feed at 64 ppm had an anti-conjugative effect. The results also suggest that AMR is reduced through other mechanisms of action, which are yet to be determined. There is insufficient evidence that flavophospholipol at 64 ppm in feed alone or with sub-therapeutic levels of antibiotics had a plasmid-curing effect.

## Introduction

Flavophospholipol (also known as bambermycin or moenomycin), is the first member of the bambermycin class of phosphoglycolipid antibiotics^1^ and exhibits bactericidal effects by inhibiting the transglycosylase activity of the penicillin-binding proteins and stopping bacterial cell wall synthesis.^2^ Flavophospholipol is primarily effective against Gram-positive bacteria, and Gram-negative bacteria such as salmonellae are only slightly susceptible.^3^ Flavophospholipol has no human therapeutic use and it has a positive effect on weight gain and feed conversion ratios in food animals.^1^ Early *in vitro* studies suggested that flavophospholipol has a ‘plasmid-curing’ effect on members of the family Enterobacteriaceae.^1^*In vitro* studies also indicated that flavophospholipol could inhibit conjugation by disrupting the formation of the plasmid bridge (pilus) between bacteria.^4^ Early *in vivo* studies showed flavophospholipol given in-feed could decrease resistance against ampicillin, tetracycline, streptomycin and/or sulphonamides in faecal *Escherichia coli* and/or salmonellae.^3^^,^^5^ However, the anti-conjugative or plasmid-curing mechanisms of action are not evident in past studies.^6^^,^^7^ There is a paucity of experimental trials validating the results *in vivo* in food animals using MDR *Salmonella*.^2^

*Salmonella* infection is a significant global public health issue, responsible for an estimated 93.8 million illnesses and 155 000 deaths annually worldwide^8^ and 1 million foodborne illnesses and more than 350 deaths annually in the United States.^9^ Poultry and contaminated poultry products are among the most frequently implicated sources.^10^ MDR *Salmonella* exacerbates the problem and is associated with more invasive and severe disease in humans.^11^ Some MDR *Salmonella* strains with extensive resistance profiles (e.g. MDR *Salmonella* Typhimurium DT104) pose greater threats to public health and have been associated with higher mortality and morbidity rates.^12^ Conjugative plasmids facilitate the movement of antimicrobial resistance (AMR) genes in different Enterobacteriaceae, including *Salmonella*.^13^ The use of agents that would not be used in human therapy and could be used to select against AMR, such as flavophospholipol, is therefore an issue of interest.

The study had two aims. The first was to determine whether flavophospholipol given in-feed at 10 ppm and 64 ppm doses could reduce conjugation of plasmid-mediated MDR *S.* Enteritidis *in vivo* in the broiler chicken model. The second aim was to determine if flavophospholipol given in-feed at 10 ppm and 64 ppm alone and in the presence of subtherapeutic levels of antimicrobials would have a plasmid-curing effect. Flavophospholipol 10 ppm is the average recommended dose^14^ and it has been demonstrated that 64 ppm could be used in broiler chickens safely.^7^

## Materials and methods

### Salmonella Enteritidis recipient and donor strains

One recipient and two donor *S.* Enteritidis strains were used in the study. The donor strain, DS1 is an R-type (AmClAxChStTeSuCeCf) that exhibits resistance to ampicillin, chloramphenicol, amoxicillin/clavulanic acid, cephalothin, streptomycin, tetracycline, sulfisoxazole, ceftriaxone and ceftiofur. The recipient strain (RS) is resistant to nalidixic acid only. A second donor strain DS2, used to test plasmid-curing, is a transconjugant (AmAxChStTeSuCpNa) that exhibits resistance to ampicillin, amoxicillin/clavulanic acid, cephalothin, streptomycin, tetracycline, sulfisoxazole, ciprofloxacin and nalidixic acid. The donor strain was a human-outbreak field isolate from the USA and the recipient strain was an environmental field isolate from Ethiopia. The MICs of selected antimicrobials for the RS and DS1 strain, as confirmed using Sensititre Gram-negative MIC plates, are shown in Table 1. Growth of mentioned strains of *S*. Enteritidis was tested on LB selective agar plates containing 128 ppm of flavophospholipol.

**Table 1.** MICs (mg/L) of antimicrobial agents for donor and recipient strains of *Salmonella* Enteritidis used in *in vitro* and *in vivo* experiments

Strain	FOX	AZM	CHL	TET	CRO	AMC	CIP	GEN	NAL	CTF	SUF	SXT	AMP	STR
Donor	>32	4	>32	>32	16	>32/16	≤0.015	0.5	4	>8	>256	≤0.12/2.38	>32	>64
Recipient	2	8	4	≤4	≤0.25	1/0.5	0.25	≤0.25	>32	1	64	>4/76	1	2

FOX, cefoxitin; AZM, azithromycin; CHL, chloramphenicol; TET, tetracycline; CRO, ceftriaxone; AMC, amoxicillin/clavulanic acid 2:1 ratio; CIP, ciprofloxacin; GEN, gentamicin; NAL, nalidixic acid; CTF, ceftiofur; SUF, sulfisoxazole; SXT, trimethoprim/sulfamethoxazole; AMP, ampicillin; STR, streptomycin.

### Conjugation assay in vitro

Conjugation experiments (using previously described procedures^15^) were performed to determine whether resistance markers were located on conjugative plasmids. Briefly, DS1 was mated with a spontaneous rifampicin-resistant, ampicillin-susceptible *E. coli* K-12 strain MG1655 on LB agar and incubated at 37°C for 6 h. The mixture was then transferred to a selective LB plate containing rifampicin and ampicillin and incubated at 37°C for 24 h. Transconjugants were purified and confirmed to be *E. coli* instead of spontaneous mutants of rifampicin-resistant *S*. Enteritidis by culturing on MacConkey agar. The second donor strain, DS2 was a transconjugant derived from conjugating DS1 and RS.

### Preparation of Salmonella Enteritidis challenge inocula

The three challenge strains (DS1, DS2 and RS) were plated onto Luria-Bertani agar (Becton Dickinson and Company, Sparks, MD, USA) and incubated at 37°C for 24 h. Growth curves were constructed for the three strains to determine the point at which cultures reached logarithmic growth. A colony from each strain was selected and transferred to three sets of 25 mL of LB broth and incubated in a shaker bath at 37°C for 4 h. Concentrations of challenge bacterial broths were adjusted to an optical density reading of 0.1 at OD_600_ with a spectrophotometer (GENESYS™ 20, Thermo Scientific, USA) which correlated to approximately 10^7^ cfu/mL of *Salmonella*, and 1.5 at OD_600_, which correlated to approximately 10^9^ cfu/mL of *Salmonella*. The concentrations of inoculum desired to test anti-conjugative effects of flavophospholipol were 10^7^ and 10^9^ cfu/mL for the donor and recipient strains of *S*. Enteritidis, respectively. Challenge concentrations were adjusted to 10^9^ cfu/mL for the strain used to test plasmid-curing effects of flavophospholipol. This is summarized in Table 2. Suspension concentrations were based on previous successful *in vivo* colonization and conjugation experiments involving *E. coli* in broiler chickens.^7^

**Table 2.** Experimental design investigating the anti-conjugative and plasmid curing effects of flavophospholipol alone and in the presence of feed grade antimicrobials on plasmid-mediated multidrug-resistant *Salmonella* Enteritidis

	Challenge group	
Intervention	*Salmonella* Enteritidis (10^7^ cfu/mL DS1 donor + 10^9^ cfu/mL RS recipient strain) testing anti-conjugative effects	*Salmonella* Enteritidis (10^9^ cfu/mL DS2 donor strain) testing plasmid curing effects
Control (non-medicated)	39 (treatment group 1)	33 (treatment group 5)
Flavophospholipol (10 ppm feed)	33 (treatment group 2)	–
Flavophospholipol (10 ppm feed) + ampicillin in water	–	33 (treatment group 6)
Flavophospholipol (64 ppm feed)	33 (treatment group 3)	33 (treatment group 7)
Flavophospholipol (64 ppm feed) + ampicillin in water	–	33 (treatment group 8)
Control (non-medicated) + ampicillin in water	33 (treatment group 4)	–

Values shown are the numbers in each group.

### Animals and housing facilities

A total of 270 female Cobb × Ross day-old chicks were obtained from two commercial local hatcheries, which are participants of the National Poultry Improvement Plan (NPIP), USA. Feed, litter and faecal samples were tested on day 0 to confirm freedom from salmonellae. Chicks were distributed and housed in 90 three-tiered cages in three separate treatment rooms with similar conditions in an approved BSL-2 animal facility in Sisson Hall, The Ohio State University. There was no direct physical contact between chicks in different cages and personal protective equipment was provided for personnel prior to entering the rooms. Chickens were given *ad libitum* access to water and a stipulated amount of feed in bowls daily. The base diet was a commercially available corn/soy-based chicken feed, which was formulated to meet or exceed the NRC recommendations, for the entire duration of the study. Approval for the project was received from the Institutional Animal Care and Use Committee at The Ohio State University, Columbus, Ohio (IACUC No. 2014A00000094).

### In vivo experimental design

Chicks were introduced into the 36 day study on day 0 and distributed into eight treatment groups (Table 2). Treatment groups 1–4 were used for testing the anti-conjugative effects and treatment groups 5–8 were used for the testing the plasmid curing effects. On day 3, chicks in treatment groups 1–4 received 0.25 mL of the donor strain DS1, and 0.25 mL of the recipient strain, RS while the chicks in treatment groups 5–8 received 0.5 mL of the DS2 transconjugant strain via oral gavage.

The eight treatment groups received one of three diets with different flavophospholipol interventions: no flavophospholipol, 10 ppm or 64 ppm from day 0 to the end of study. Flavophospholipol was supplied as the commercially available, FDA-approved Flavomycin 4^®^ (Huvepharma^®^, NADA #44-759). Flavomycin contains the active ingredient (flavophospholipol) at a concentration of 8.8 g/kg (4 g/lb). The feed was mixed in-house in accordance with the manufacturer’s instructions. Subtherapeutic levels of ampicillin were given in water to treatment groups 4, 6 and 8 for 12 days to test the application of selective pressure and promotion of AMR. Chickens were provided 100 mg/kg of live body weight or half doses of ampicillin for 12 days from day 10 to 21. Doses were prepared for an average of 270 g in live body weight from day 10 to 15 and 650 g in live body weight from day 16 to 21. Stock solutions were made from ampicillin trihydrate (Fisher Scientific, USA) and diluted to the required concentrations. Fresh solutions were prepared for the treatment groups daily.^2^

Cloacal samples were collected at 4, 11 and 20 days post inoculation (p.i.) and caecal samples from euthanized chickens were collected at the end of the study (32 days p.i.).

### Bacteriological methods

*Salmonella* was isolated by conventional methods.^16^ Briefly, samples were placed in buffered peptone water (Becton Dickinson and Company, Sparks, MD, USA) at approximately 1 : 10 ratio and incubated at 37°C for 24 h. An aliquot of 100 μL of the broth was transferred to 9.9 mL of Rappaport-Vassiliadis broth (Oxoid, Hants, England) and incubated for 18 to 24 h at 42°C. The broth was streaked onto selective Xylose-Lysine-Tergitol 4 (XLT4) agar plates (Becton Dickinson and Company, Sparks, MD, USA) with and without antibiotics and incubated for 18 to 24 h. All antibiotic stocks used for the making of XLT4 plates with antibiotics were prepared according to manufacturer’s specifications (Sigma Aldrich, St Louis, MO, USA). Samples from chickens in treatment groups 1–4 were streaked on selective XLT4 agar plates containing 100 ppm ampicillin and 30 ppm nalidixic acid to select for transconjugants which were naturally resistant to nalidixic acid and acquired resistance to ampicillin. Samples from chickens in treatment groups 5–8 were streaked on selective XLT4 agar plates with 30 ppm nalidixic acid to select for nalidixic acid-resistant transconjugants. Up to three colonies per positive sample were isolated and cryopreserved for phenotypic characterization. Conjugation events between donor and recipient strains were only confirmed in chickens in treatment groups 1–4 if transconjugant salmonellae could be isolated from their cloacal or caecal samples.

### Antimicrobial susceptibility testing

Antimicrobial susceptibility testing was done with a panel of 12 antimicrobials using the Kirby-Bauer method.^17^ The following are the BD BBL™ Sensi-Disc™ (Becton Dickinson and Company, Sparks, MD, USA) antimicrobial susceptibility test discs used with their respective disc potencies: ampicillin (Am, 10 μg), tetracycline (Te, 30 μg), ceftiofur (XNL, 30 μg), chloramphenicol (C, 30 μg), sulfisoxazole (G, 250 μg), streptomycin (S, 10 μg), kanamycin (K, 30 μg), gentamicin (CN, 10 μg), ceftriaxone (CRO, 30 μg), ciprofloxacin (CIP, 5 μg), cephalothin (KF, 30 μg) and amoxicillin/clavulanic acid (AMC, 30 μg). Zone diameter sizes were evaluated according to CLSI breakpoints. *E. coli* ATCC 25922, *Enterococcus faecalis* ATCC 29212, *Staphylococcus aureus* ATCC 25923 and *Pseudomonas aeruginosa* ATCC 27853 were used as quality control organisms.

### Molecular analysis

PFGE was performed on DS1, DS2, and RS to determine if the isolates were from the challenge strains or any extraneous *Salmonella* introduced during the study. At each timepoint, we randomly selected two isolates (from two different chickens) per group for analysis. PFGE was performed according to CDC’s PulseNet protocol.^18^ DNA was digested with 50 U of XbaI restriction enzyme (New England Biolabs, Ipswich, MA, USA) for at least 2 h at 37°C. *Salmonella* Braenderup H9812 was used as a molecular reference marker. Electrophoresis was performed using CHEF-DR^®^ III Pulsed-Field Electrophoresis System (Bio-Rad Laboratories, Hercules, CA, USA) with the following conditions and reagents: 1% SeaKem Gold agarose (FMC BioProducts, Rockland, Maine, USA) in 0.5% Tris-borate EDTA buffer, temperature: 14°C; voltage: 6 V/cm; run time: 18 h with switch times ranging from 2.2 to 63.8 s. The gels were stained with ethidium bromide and the DNA bands were visualized under UV trans-illumination (Gel Doc™ 2000, Bio-Rad Laboratories, Hercules, CA, USA) and gel images were captured using the Quantity one 1-D analysis software (Bio-Rad Laboratories, Hercules, CA, USA). PFGE gels were consolidated and visualized using Bionumerics software V. 4.61 (Applied Maths NV, Belgium).

### Statistical analysis

The statistical software used was Intercooled STATA 12 (StataCorp, College Station, TX). Correlation of observations of chickens within a cage were accounted for in analyses and sample size determinations. Sample size calculations were made using the module ‘*clustersampsi*’.^19^ It was assumed that 80% of isolates in poultry would be resistant post-inoculation as studies in organic and conventional broiler farms show that resistance ranged from 55.2% to 91.4%.^20^ An absolute decrease of 50% was assumed based on earlier studies of flavophospholipol showing that it significantly reduced the detection of *Salmonella* with MDR phenotypes by about 40%^6^ and the mean degree of ampicillin resistance in *E. coli* by about 64%.^3^ To detect an absolute decrease of 50% in AMR with 80% power, with a significance level of 5%, and assuming a fixed number of three chickens per cage, 30 chickens per intervention group were required. Allowing for a 10% mortality rate in chickens, we planned for 33 chickens per intervention group except for the control group 1, which had 39 chickens to account for additional mortalities due to the absence of flavophospholipol. The sample size calculation assumed an Intracluster Correlation (ICC) of 0.5–0.6 based on previous estimates of ICC in AMR of isolates from the same animal.^21^ This is a conservative estimate of the ICC for our study since we expect within-cage ICCs to be smaller than within-chicken ICCs. A *P* value <0.05 was considered statistically significant.

Differences in the proportions of chickens testing positive for transconjugant salmonella among groups 1–4 were assessed using logistic regression with robust standard errors accounting for within-cage relationships [*vce* (*cluster*) option in the logistic command in STATA^22^]. Separate models were fit to data at each timepoint and Bonferroni corrections were used to control the overall type-I error rate across pairwise comparisons of treatment groups. Differences in proportions of nalidixic acid-resistant salmonellae that either retained or lost ampicillin resistance (binary variable) among groups 5–8 were also assessed using logistic regression with robust standard errors.

## Results

**Figure 1.**
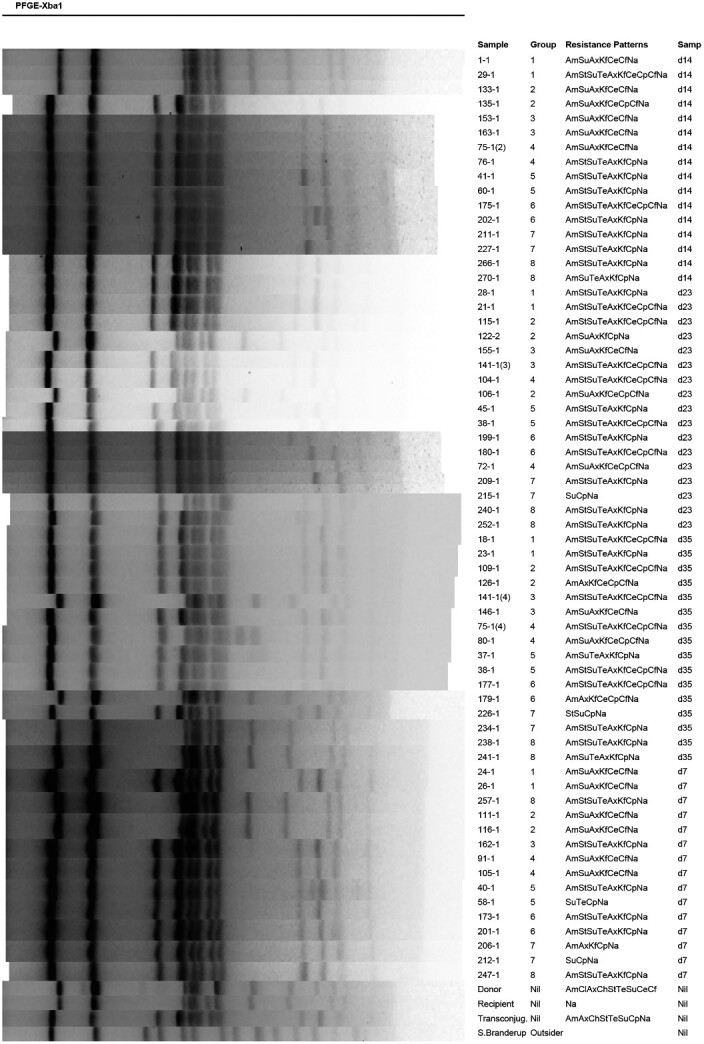
PFGE dendrogram of DS, RS1, RS2 and selected isolates from treatment groups. The selected isolates were clonally related to the challenge strains and no other extraneous salmonellae were detected.

A total of 2137 *Salmonella* isolates were tested for antimicrobial susceptibility and 66 isolates showed changes in AMR profiles. The 66 isolates along with the three challenge strains were collected and processed for DNA fingerprinting by PFGE to determine clonality of the isolates (Figure 1). There were no significant acquisitions of resistances to chloramphenicol, gentamicin and kanamycin by the recipient strain (RS) and transconjugant strain (DS2). The differences in resistances to the following antimicrobials were assessed in detail: ampicillin, streptomycin, tetracycline, ceftriaxone and ceftiofur.

### Lower numbers of chickens with ampicillin-resistant salmonellae were observed in Treatment Group 3 (flavophospholipol 64 ppm)

**Figure 2.**
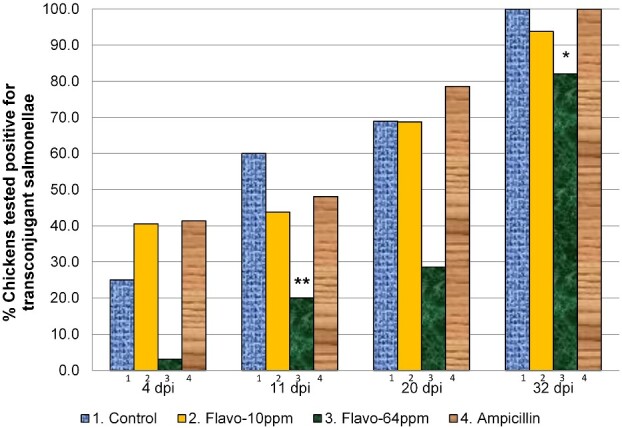
Proportion of chickens from which ampicillin-resistant transconjugant salmonellae could be recovered in treatment groups 1–4. Key: *, proportions that were marginally significantly different from the control (*P < *0.1); **, proportions that were significantly different from the control (*P < *0.05).

The proportion of chickens that tested positive for ampicillin-resistant salmonellae in treatment groups 1–4 are shown in Figure 2 and Table 3. A greater proportion of chickens in the control group had ampicillin-resistant isolates than in treatment group 3 (flavophospholipol 64 ppm) at 11 days p.i. (OR 5.99, 95% CI 1.79–20.00, *P = *0.036). There were no observable differences in the number of chickens that tested positive for ampicillin-resistant transconjugants between the control group and treatment group 4, which was given sub-therapeutic levels of ampicillin.

**Table 3.** Numbers and OR of chickens which tested positive (R+) and tested negative (R−) for ampicillin-resistant transconjugant salmonellae

Timepoint	Treatment	R−	R+	% R+	OR (95% CI)	Bonferroni corrected *P* value
4 days p.i.	Control	27	9	25.0	reference	
	Flavo-10 ppm	19	13	40.6	2.05 (0.65–6.53)	1.000
	Flavo-64 ppm	31	1	3.1	0.10 (0.01–0.77)	0.243
	Ampicillin	17	12	41.4	2.12 (0.61–7.36)	1.000
11 days p.i.	Control	14	21	60.0	reference	
	Flavo-10 ppm	18	14	43.8	0.52 (0.17–1.60)	1.000
	Flavo-64 ppm	24	6	20.0	0.17 (0.05–0.56)	0.036
	Ampicillin	14	13	48.1	0.62 (0.19–2.00)	1.000
20 days p.i.	Control	11	21	69.0	reference	
	Flavo-10 ppm	10	22	68.8	1.15 (0.37–3.56)	1.000
	Flavo-64 ppm	20	8	28.6	0.21 (0.06–0.76)	0.162
	Ampicillin	6	22	78.6	1.92 (0.43–8.56)	1.000
32 days p.i.	Control	0	32	100.0	reference	
	Flavo-10 ppm	2	30	93.8	0.43 (0–5.66)	1.000 (exact)
	Flavo-64 ppm	5	23	82.1	0.35 (0–0.97)	0.064 (exact)
	Ampicillin	0	27	100	–	–

Robust standard errors (SEs), accounting for cage clustering were used. Bonferroni correction of 9 was used for comparisons of swab results (3 timepoints × 3 treatments compared with control) and a correction of 3 was used to compare caecum results at 32 days p.i. (3 treatments compared with control). Exact logistic regression models were used where (exact) is indicated.

### Reduced acquisition of resistance to streptomycin and tetracycline by salmonellae in Treatment Group 3 (flavophospholipol 64 ppm)

**Figure 3.**
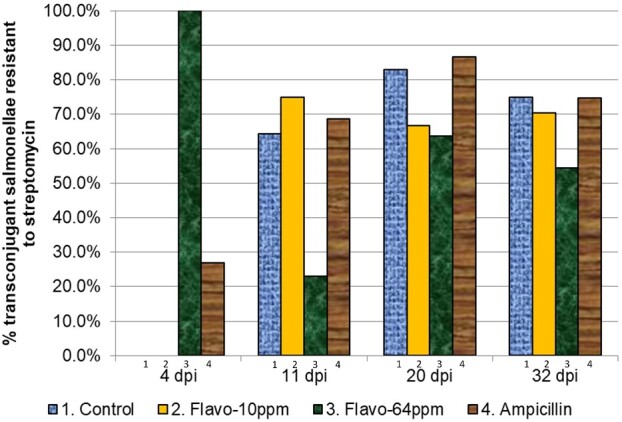
Proportion of ampicillin-resistant transconjugant salmonellae isolated from treatment groups 1–4 that were also resistant to streptomycin.

**Figure 4.**
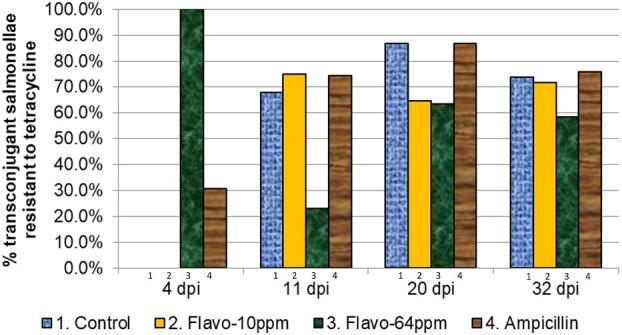
Proportion of ampicillin-resistant transconjugant salmonellae isolated from treatment groups 1–4 that were also resistant to tetracycline.

Streptomycin and tetracycline resistance in ampicillin-resistant salmonellae isolated from treatment groups 1–4 are shown in Figure 3 and Figure 4, and Table 4 and Table 5, respectively. Reduced acquisition of resistance to streptomycin and tetracycline in treatment group 3 as compared with the control was observed at 11, 20 and 32 days p.i., although this reduction was not statistically significant. The resistance pattern of the transconjugants to streptomycin and tetracycline in treatment group 2 was similar to that in the control. There were no observable differences in extended-spectrum cephalosporin resistance (ESC-R) in isolates between each treatment and control group (data not shown).

**Table 4.** Proportion of streptomycin-resistant transconjugants in treatment groups 1–4

Timepoint	Treatment	R−	R+	% R+
4 days p.i.	1. Control	17	0	0
	2. Flavo-10 ppm	23	0	0
	3. Flavo-64 ppm	0	1	100.0
	4. Ampicillin	19	7	26.9
11 days p.i.	1. Control	21	38	64.4
	2. Flavo-10 ppm	6	18	75.0
	3. Flavo-64 ppm	10	3	23.1
	4. Ampicillin	11	24	68.6
20 days p.i.	1. Control	9	44	83.0
	2. Flavo-10 ppm	18	36	66.7
	3. Flavo-64 ppm	8	14	63.6
	4. Ampicillin	8	52	86.7
32 days p.i.	1. Control	22	66	75.0
	2. Flavo-10 ppm	26	62	70.5
	3. Flavo-64 ppm	32	38	54.3
	4.Ampicillin	19	56	74.7

R, streptomycin resistance.

**Table 5.** Proportion of tetracycline-resistant transconjugants in treatment groups 1–4

Timepoint	Treatment	R−	R+	% R+
4 days p.i.	1. Control	17	0	0.0
	2. Flavo-10 ppm	23	0	0.0
	3. Flavo-64 ppm	0	1	100.0
	4. Ampicillin	18	8	30.8
11 days p.i.	1. Control	19	40	67.8
	2. Flavo-10 ppm	6	18	75.0
	3. Flavo-64 ppm	10	3	23.1
	4. Ampicillin	9	26	74.3
20 days p.i.	1. Control	7	46	86.8
	2. Flavo-10 ppm	19	35	64.8
	3. Flavo-64 ppm	8	14	63.6
	4. Ampicillin	8	52	86.7
32 days p.i.	1. Control	23	65	73.9
	2. Flavo-10 ppm	25	63	71.6
	3. Flavo-64 ppm	29	41	58.6
	4. Ampicillin	18	57	76.0

R, tetracycline resistance.

### No loss of resistance in MDR salmonellae in chickens given in-feed flavophospholipol alone and with sub-therapeutic levels of ampicillin

**Figure 5.**
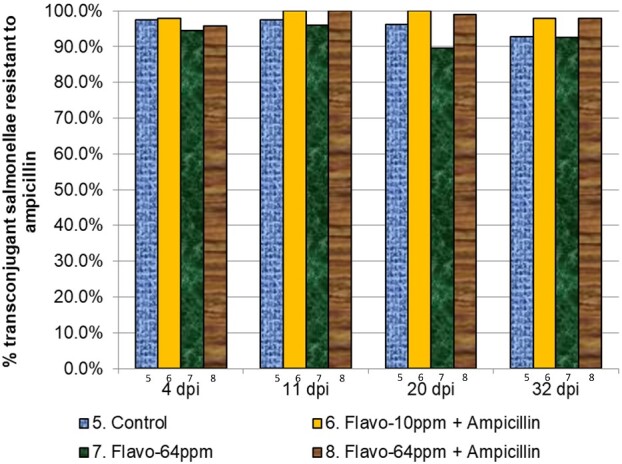
Proportion of transconjugants in treatment groups 5–8 which were resistant to ampicillin. There was no significant differences in ampicillin resistance between treatment groups (*P > *0.1).

The prevalence and associated loss of plasmid-mediated ampicillin resistance did not differ between treatment groups 5–8 (Figure 5 and Table 6). There were no clear, observable differences in resistances to streptomycin and tetracycline (data not shown).

**Table 6.** Comparison of ampicillin-resistant transconjugants in treatment groups 6–8 versus control group 5

Timepoint	Treatment	R−	R+	% R+	OR (95% CI)	Bonferroni corrected *P* value
4 days p.i.	5. Control	2	80	97.6	reference	–
	6. Flavo-10 ppm + AMP	2	90	97.8	1.13 (0.20–6.26)	1.000
	7. Flavo-64 ppm	5	85	94.4	0.43 (0.11–1.62)	1.000
	8. Flavo-64 ppm + AMP	4	93	95.9	0.58 (0.12–2.82)	1.000
11 days p.i.	5. Control	2	78	97.5	reference	–
	6. Flavo-10 ppm + AMP	0	95	100.0	2.89 (0.22 to ∞)	1.000 (exact)
	7. Flavo-64 ppm	3	73	96.1	0.62 (0.13–3.09)	1.000
	8. Flavo-64 ppm + AMP	0	98	100.0	1.44 (0.61 to ∞)	1.000 (exact)
20 days p.i.	5. Control	3	77	96.3	reference	–
	6. Flavo-10 ppm + AMP	0	91	100.0	4.47 (0.47 to ∞)	0.900 (exact)
	7. Flavo-64 ppm	9	77	89.5	0.33 (0.08–1.37)	1.000
	8. Flavo-64 ppm + AMP	1	89	98.9	3.47 (0.39–31.08)	1.000
32 days p.i.	5. Control	6	78	92.9	reference	–
	6. Flavo-10 ppm + AMP	2	96	98.0	3.69 (0.47–28.71)	0.636
	7. Flavo-64 ppm	7	86	92.5	0.95 (0.31–2.88)	1.000
	8. Flavo-64 ppm + AMP	2	92	97.9	3.54 (0.82–15.26)	0.270

R, ampicillin resistance; AMP, ampicillin. Robust standard errors (SEs), accounting for cage clustering were used. A Bonferroni correction of 9 was used for timepoints 4, 11 and 20 days post inoculation, to control errors introduced in pair-wise tests comparing swab results in 3 treatment groups and control, and a Bonferroni correction of 3 was applied at timepoint 32 days post inoculation, to control errors introduced in pair-wise tests comparing caecum results in 3 treatment groups and control. Exact logistic regression models were used where (exact) is indicated.

### Reduced acquisition of resistance to third-generation cephalosporins with in-feed flavophospholipol 64 ppm

**Figure 6.**
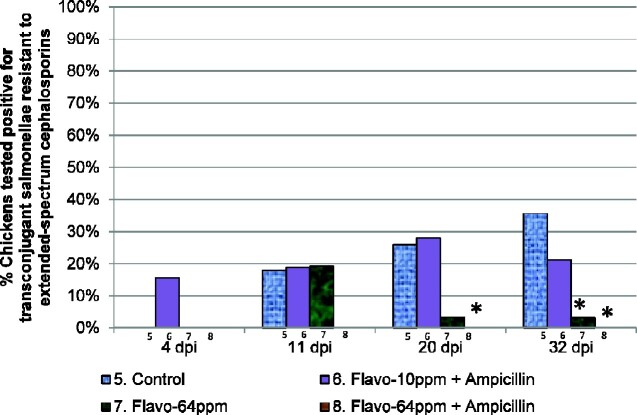
Proportion of chickens in treatment groups 5–8 from which transconjugant salmonellae resistant to extended-spectrum cephalosporins were recovered. Key: *, proportions that were marginally significantly different from the control (*P < *0.1); **, proportions that were significantly different from the control (*P < *0.05).

A higher proportion of chickens with ESC-R transconjugant salmonellae in the control group was observed compared with treatment group 8 (flavophospholipol 64 ppm and ampicillin) at 20 days p.i. (OR 2.40, 95% CI 1.25 to ∞, *P = *0.053) (Figure 6 and Table 7). A significantly higher proportion of chickens with ESC-R transconjugant salmonellae were recovered in the control group as compared with treatment group 7 (flavophospholipol 64 ppm) at 32 days p.i. (OR 16.67, 95% CI 2.02–142.86, *P = *0.027) and as compared with treatment group 8 at 32 days p.i. (OR 2.84, 95% CI 1.50 to ∞, *P = *0.0009). This was indicative of reduced acquisition of ESC-R by DS2 in chickens in groups given in-feed flavophospholipol 64 ppm.

**Table 7.** Comparison of chickens which tested positive (R+) and number of chickens which tested negative (R−) for transconjugant salmonellae that acquired resistance to extended-spectrum cephalosporins

Timepoint	Treatment	R−	R+	% R+	OR (95% CI)	Bonferroni corrected *P* value
4 days p.i.	5. Control	29	0	0	reference	
	6. Flavo-10 ppm + AMP	27	5	15.6	6.84 (0.88–∞)	0.4842 (exact)
	7. Flavo-64 ppm	32	0	0	–	–
	8. Flavo-64 ppm + AMP	34	0	0	–	–
11 days p.i.	5. Control	23	5	17.9	reference	
	6. Flavo-10 ppm + AMP	26	6	18.8	1.06 (0.19–5.92)	1.000
	7. Flavo-64 ppm	21	5	19.2	1.10 (0.29–4.12)	1.000
	8. Flavo-64 ppm + AMP	32	0	0	0.48 (0–0.96)	0.324 (exact)
20 days p.i.	5. Control	20	7	25.9	reference	
	6. Flavo-10 ppm + AMP	23	9	28.1	1.12 (0.29–4.26)	1.000
	7. Flavo-64 ppm	29	1	3.3	0.10 (0.01–0.80)	0.279
	8. Flavo-64 ppm + AMP	31	0	0	0.42 (0–0.80)	0.053 (exact)
32 days p.i.	5. Control	18	10	35.7	reference	
	6. Flavo-10 ppm + AMP	26	7	21.2	0.48 (0.08–2.82)	1.000
	7. Flavo-64 ppm	30	1	3.2	0.06 (0.01–0.50)	0.027
	8. Flavo-64 ppm + AMP	32	0	0	0.35 (0–0.67)	0.0009 (exact)

AMP, ampicillin. Robust standard errors (SEs), accounting for cage clustering were used. A Bonferroni correction of 9 was used for timepoints 4, 11 and 20 days post inoculation, to control errors introduced in pair-wise tests comparing swab results in 3 treatment groups and control, and a Bonferroni correction of 3 was applied at timepoint 32 days post inoculation, to control errors introduced in pair-wise tests comparing caecum results in 3 treatment groups and control. Exact logistic regression models were used where (exact) is indicated.

**Figure 7.**
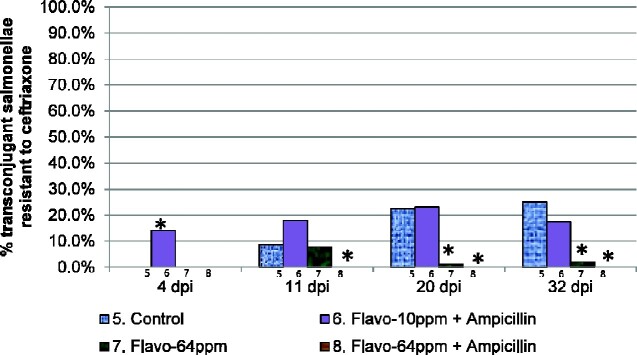
Proportion of transconjugants in treatment groups 5–8 which were resistant to ceftriaxone. Key: *, proportions that were marginally significantly different from the control (*P < *0.1); **, proportions that were significantly different from the control (*P < *0.05).

**Figure 8.**
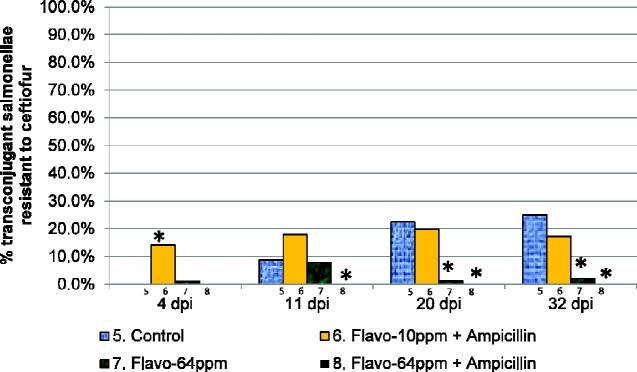
Proportion of transconjugants in treatment groups 5–8 which were resistant to ceftiofur. * indicates proportions that were marginally significantly different from the control (*P < *0.1). ** indicates proportions that were significantly different from the control (*P < *0.05).

The proportion of nalidixic acid-resistant transconjugant salmonellae that acquired resistance to ceftriaxone and ceftiofur across treatment groups 5–8 is shown in Figure 7 and Figure 8 and Table 8 and Table 9, respectively. Significantly greater proportions of ESC-R nalidixic acid-resistant salmonellae were recovered from the control group as compared with those from treatment group 7 at 20 days p.i. (OR 24.39, 95% CI 2.86–208.33, *P = *0.027) and 32 days p.i. (OR 15.15, 95% CI 2.00–114.9, *P = *0.027). Similarly, greater proportions of nalidixic acid-resistant salmonellae recovered from control group 5 were ESC-R as compared with those from treatment group 8 at the 20 days p.i. (OR 3.33, 95% CI 1.81 to ∞, *P < *0.0001) and at 32 days p.i. timepoints (OR 3.57, 95% CI 1.96 to ∞, *P < *0.0001).

**Table 8.** Proportions and odds of ceftriaxone-resistant transconjugants in treatment groups 6–8 versus control group 5, accounting for cage clustering

Timepoint	Treatment	R−	R+	% R+	OR (95% CI)	Bonferroni corrected *P* value
4 days p.i.	5. Control	82	0	0.0	reference	–
	6. Flavo-10 ppm + AMP	79	13	14.1	18.57 (3.01 to ∞)	0.0027 (exact)
	7. Flavo-64 ppm	90	0	0.0	–	–
	8. Flavo-64 ppm + AMP	97	0	0.0	–	–
11 days p.i.	5. Control	73	7	8.8	reference	–
	6. Flavo-10 ppm + AMP	78	17	17.9	2.27 (0.41–12.46)	1.000
	7. Flavo-64 ppm	70	6	7.9	0.89 (0.25–3.20)	1.000
	8. Flavo-64 ppm + AMP	98	0	0.0	0.43 (0–0.81)	0.0576 (exact)
20 days p.i.	5. Control	62	18	22.5	reference	–
	6. Flavo-10 ppm + AMP	70	21	23.1	1.03 (0.26–4.13)	1.000
	7. Flavo-64 ppm	85	1	1.2	0.041 (0.0048–0.35)	0.027
	8. Flavo-64 ppm + AMP	90	0	0.0	0.30 (0–0.55)	<0.0001 (exact)
32 days p.i.	5. Control	63	21	25.0	reference	–
	6. Flavo-10 ppm + AMP	81	17	17.3	0.63 (0.13–3.09)	1.000
	7. Flavo-64 ppm	91	2	2.2	0.07 (0.0087–0.50)	0.027
	8. Flavo-64 ppm + AMP	94	0	0.0	0.28 (0–0.51)	<0.0001 (exact)

R, ceftriaxone resistance; AMP, ampicillin. Robust SEs, accounting for cage clustering were used for timepoints 4, 11 and 20 days post inoculation (p.i.). A Bonferroni correction of 9 was used for these timepoints, to control errors introduced in pair-wise tests comparing swab results in 3 treatment groups and control, and a Bonferroni correction of 3 was applied at timepoint 32 days p.i., to control errors introduced in pair-wise tests comparing caecum results in 3 treatment groups and control. Exact logistics regression models were used where (exact) is indicated.

**Table 9.** Proportions and odds of ceftiofur-resistant transconjugants in treatment groups 6–8 versus control group 5, accounting for cage clustering

Timepoint	Treatment	R−	R+	% R+	OR (95% CI)	Bonferroni corrected P-value
4 days p.i.	5. Control	82	0	0.0	reference	–
	6. Flavo-10 ppm + AMP	79	13	14.1	18.57 (3.01 to ∞)	0.0027 (exact)
	7. Flavo-64 ppm	89	1	1.1	0.95 (0.15 to ∞)	1.000 (exact)
	8. Flavo-64 ppm + AMP	97	0	0.0	–	–
11 days p.i.	5. Control	73	7	8.8	reference	–
	6. Flavo-10 ppm + AMP	78	17	17.9	2.27 (0.58–8.98)	1.000
	7. Flavo-64 ppm	70	6	7.9	0.89 (0.22–3.56)	1.000
	8. Flavo-64 ppm + AMP	98	0	0.0	0.43 (0–0.81)	0.0576 (exact)
20 days p.i.	5. Control	62	18	22.5	reference	–
	6. Flavo-10 ppm + AMP	73	18	19.8	0.85 (0.19–3.87)	1.000
	7. Flavo-64 ppm	85	1	1.2	0.041 (0.0048–0.35)	0.027
	8. Flavo-64 ppm + AMP	90	0	0.0	0.30 (0–0.55)	<0.0001 (exact)
32 days p.i.	5. Control	63	21	25.0	reference	–
	6. Flavo-10 ppm + AMP	81	17	17.3	0.63 (0.13–3.09)	1.000
	7. Flavo-64 ppm	91	2	2.2	0.07 (0.0087–0.50)	0.027
	8. Flavo-64 ppm + AMP	94	0	0.0	0.28 (0–0.51)	<0.0001 (exact)

R, ceftiofur resistance; AMP, ampicillin. Robust SEs, accounting for cage clustering were used for timepoints 4, 11 and 20 days post inoculation (p.i.) A Bonferroni correction of 9 was used for timepoints 4, 11 and 20 days p.i., to control errors introduced in pair-wise tests comparing swab results in 3 treatment groups and control, and a Bonferroni correction of 3 was applied at timepoint 32 days p.i., to control errors introduced in pair-wise tests comparing caecum results in 3 treatment groups and control. Exact logistics regression models were used where (exact) is indicated.

## Discussion

Ampicillin-resistant *Salmonella* transconjugants that resulted from *in vivo* conjugation were recovered from treatment groups 1–4. There were significantly reduced numbers of chickens that tested positive for ampicillin-resistant transconjugant salmonellae in treatment group 3 (flavophospholipol 64 ppm) as compared with the control group 1 across time, indicative of reduced acquisition of ampicillin resistance in chickens given flavophospholipol 64 ppm. Anti-conjugative effects were significant at 11 days p.i. and marginally significant at 32 days p.i. Results were similar but not significant at 4 and 20 days p.i. The findings in this study indicated that the use of in-feed flavophospholipol at 64 ppm resulted in the reduction of conjugation events. This is consistent with previously hypothesized anti-conjugative effects of flavophospholipol, but the effect was only seen with flavophospholipol given at higher than the recommended dose of 10 mg/kg. Further studies to determine dose–response relationships and the effects of graduated doses of flavophospholipol and its anti-conjugative effects would be needed to determine the ideal concentration to achieve maximum effect.

Streptomycin and tetracycline resistance was not determined *in vitro* to be carried on conjugative plasmids prior to the study. However, the ampicillin-resistant transconjugants recovered in treatment groups 1–4 had also acquired resistance to tetracycline and streptomycin. There was reduced resistance to streptomycin and tetracycline in the treatment group given flavophospholipol in-feed at 64 ppm compared with the control. While inconclusive on its own, this finding suggests that transconjugant salmonellae in chickens in treatment group 3 did not acquire resistance to streptomycin and tetracycline at the same rate as that in the control group.

The loss of plasmid-mediated ampicillin resistance or plasmid curing was not significant with the addition of flavophospholipol in-feed at 64 ppm alone or with subtherapeutic levels of ampicillin in groups 5–8.

A significant observation was that the transconjugants in treatment groups 5–8 unexpectedly acquired ESC-R. While poultry production rarely uses cephalosporin treatments, broiler chickens could harbour ESBL or pAmpC-producing *E. coli*.^18^ These chickens could be colonized with commensal bacteria or *E. coli* harbouring plasmids conferring resistance to extended-spectrum cephalosporins. This was not evident in groups 1–4 due to possible co-selection of other antibiotic resistance such as streptomycin or tetracycline, associated with the transfer of other mobile genetic elements.^23^

When we compared the proportion of chickens in treatment groups 5–8 that tested positive for ESC-R transconjugants, a significantly greater proportion of chickens in control group 5 tested positive for these transconjugants than in treatment groups 7 and 8 at various timepoints. None of the isolates in treatment group 8 (flavophospholipol 64 ppm and ampicillin) displayed ESC-R. Flavophospholipol reduces the acquisition of ESC-R.

There was insufficient evidence to determine whether subtherapeutic doses of ampicillin increased conjugation events in the chicken gut. It is likely that long-term and low-level exposures to antimicrobials would exert selection pressure as opposed to short-term, full or subtherapeutic dosing.^24^ Thus, the impact of subtherapeutic levels of ampicillin is more observable in a commercial poultry farm setting, with multiple production cycles.

The transconjugants isolated across all treatment groups and times exhibited phenotypes with multiresistance. A majority acquired resistance to cephalothin, a first-generation cephalosporin antibiotic, and amoxicillin/clavulanic acid. This was previously suggested to be caused by an over-expression of the *bla*_PSE1_ gene, which encodes resistance to ampicillin and amoxicillin/clavulanic acid.^25^ As the majority of transconjugants were resistant to ampicillin, cross-resistance to cephalothin and amoxicillin/clavulanic acid is expected. Acquisition of ciprofloxacin resistance in the transconjugants is also observed. This could be due to an associated nalidixic acid resistance, which is mediated by quinolone resistance-determining regions of *gyrA*.^26^

Only ampicillin resistance was confirmed to be carried on a conjugative plasmid prior to the study. Additional steps are needed to determine whether resistance to streptomycin, tetracycline and extended-spectrum cephalosporins are plasmid-mediated. Flavophospholipol could alter the microbiome or bacterial community of the chicken gut to a state that is not ideal for the transfer of resistance between bacteria or which promotes the excretion of MDR bacteria. Plasmid profile analysis of *Salmonella* isolates from the study and metagenomic studies of the gut of chickens in different intervention groups would be useful. Further *in vivo* studies to determine dose–response relationships of flavophospholipol will be helpful to determine an ideal concentration for animal production.

### Conclusions

This study has shown that flavophospholipol given in-feed at 64 ppm has anti-conjugative effects *in vivo* in the chicken gut. In a farm setting, flavophospholipol could have the potential to reduce the transmission of plasmid-mediated AMR between animals and have a positive effect in minimizing the risk of AMR transmission to humans through both direct contact and foodborne routes. There was insufficient evidence that flavophospholipol in-feed alone or with subtherapeutic levels of ampicillin had any plasmid-curing effect. Flavophospholipol has no analogue used for human therapy. This advantage coupled with the results from this study that flavophospholipol has anti-conjugative properties make it an attractive feed additive. It may be necessary to review the dose limit to consider maximizing the effect of flavophospholipol in reducing the acquisition of AMR.
